# Do Ravens Show Consolation? Responses to Distressed Others

**DOI:** 10.1371/journal.pone.0010605

**Published:** 2010-05-12

**Authors:** Orlaith N. Fraser, Thomas Bugnyar

**Affiliations:** 1 Department of Cognitive Biology, University of Vienna, Vienna, Austria; 2 Konrad Lorenz Forschungstelle, Grünau, Austria; Georgia State University, United States of America

## Abstract

**Background:**

Bystander affiliation (post-conflict affiliation from an uninvolved bystander to the conflict victim) may represent an expression of empathy in which the bystander consoles the victim to alleviate the victim's distress (“consolation”). However, alternative hypotheses for the function of bystander affiliation also exist. Determining whether ravens spontaneously offer consolation to distressed partners may not only help us to understand how animals deal with the costs of aggressive conflict, but may also play an important role in the empathy debate.

**Methodology/Principal findings:**

This study investigates the post-conflict behavior of ravens, applying the predictive framework for the function of bystander affiliation for the first time in a non-ape species. We found weak evidence for reconciliation (post-conflict affiliation between former opponents), but strong evidence for both bystander affiliation and solicited bystander affiliation (post-conflict affiliation from the victim to a bystander). Bystanders involved in both interactions were likely to share a valuable relationship with the victim. Bystander affiliation offered to the victim was more likely to occur after intense conflicts. Renewed aggression was less likely to occur after the victim solicited affiliation from a bystander.

**Conclusions/Significance:**

Our findings suggest that in ravens, bystanders may console victims with whom they share a valuable relationship, thus alleviating the victims' post-conflict distress. Conversely victims may affiliate with bystanders after a conflict in order to reduce the likelihood of renewed aggression. These results stress the importance of relationship quality in determining the occurrence and function of post-conflict interactions, and show that ravens may be sensitive to the emotions of others.

## Introduction

Aggressive conflicts feature regularly in the lives of many group-living animals over matters such as positions in the dominance hierarchy, access to limited resources, or over decisions that have to be made. Such conflicts, however, may be costly, using up valuable energy and time and risking injury. Moreover, aggressive conflicts may damage the opponents' relationship [1], [2], leading to a loss of benefits afforded by that relationship such as food-sharing or support in future conflicts, and opponents may become distressed [3], [4]. One way of reducing the costs of aggressive conflict is through reconciliation, the post-conflict affiliative reunion between former opponents [5], which has been shown to repair the opponents' relationship and alleviate post-conflict distress [4]. However, approaching a former opponent so soon (usually within the first ten minutes) after a fight carries risks of renewed aggression and so reconciliation is only likely to occur when the benefits outweigh the costs [4], [6]. When de Waal & van Roosmalen [5] first described reconciliation in chimpanzees in 1979, they also described bystanders uninvolved in the preceding conflict embracing the victim once the conflict ceased, a phenomenon they labeled ‘consolation’, as it was presumed to alleviate the victim's distress. Consolation is a particularly interesting interaction because it implies a cognitively demanding degree of empathy, known in humans as ‘sympathetic concern’ [7]. In order for a bystander to console a victim, they must first recognize that the victim is distressed and then act appropriately to alleviate that distress, requiring a sensitivity to the emotional needs of others previously attributed only to humans. Indeed, the apparent absence of consolation in monkeys has been suggested to result from their lack of the requisite degree of empathy for consolation to occur [8]. Concordantly, Japanese macaque mothers of distressed conflict victims, in a situation when an empathic response would be most expected, showed no signs of distress themselves and made no attempt to console their offspring [9]. However, the degree to which apes, monkeys and indeed all other animals are capable of empathy is still a matter of debate [7], [10]–[18]. Although very little is known about empathy in birds, a recent study on graylag geese has shown that bystanders who observed a conflict involving either their pair partner or a family member experienced an increase in heart-rate (a measure of distress) indicative of an empathic response [19].

The term consolation implies a distress-alleviating function and a motivation rooted in empathy for the distressed victim but post-conflict affiliation from bystanders to victims (hereafter ‘bystander affiliation’ when no functional or mechanistic assumptions are made; Table 1) may not always be consoling. Indeed, of the two studies to investigate consolation in the functional sense, only was able to find a distress-alleviating effect (evidence against: [20]; evidence for: [21]). Furthermore, suggestive evidence is available for two alternative functions of bystander affiliation, for which empathy is not required.

**Table 1:** Definitions of terms used.

Term	Definition
Bystander affiliation	Post-conflict affiliative interaction initiated by a bystander and directed towards the conflict victim. No functional or mechanistic implications. Also known as (unsolicited) triadic or third-party affiliation.
Solicited bystander affiliation	Post-conflict affiliation interaction initiated by the conflict victim and directed towards a bystander.
Consolation	Bystander affiliation that serves to alleviate the victim's distress. Implies that consolers are motivated by empathy for the victim.

After aggressive conflicts, the kin of both the aggressor and the victim may be more likely to affiliate with their relative's opponent [22], [23]. Thus, bystander affiliation may in fact be a form of kin-mediated reconciliation, which may enable the opponents' relationship to be repaired without risking renewed aggression by directly approaching an opponent soon after the cease of aggressive conflict. Accordingly, friendly grunts from the aggressor's kin to the victim have been shown to restore tolerance between former opponents in savannah baboons [24]. In species with strong relationships between non-kin, unrelated valuable partners may also be able to act as a proxy for the aggressor in reconciling with the victim of aggression, as bystanders who initiated post-conflict affiliation with the victim were found to have more valuable relationships with the aggressor than with the victim in chimpanzees [25].

Bystander affiliation has also been suggested to function as a mechanism to protect the bystander from becoming a target of redirected aggression [26], [27]. Redirected aggression is defined as post-conflict aggression from the original victim to a bystander, which may reverse the negative consequences of losing the original conflict [28], [29]. Accordingly, in a population of captive chimpanzees, those who provided bystander affiliation to the victim were found to be those most at risk from redirected aggression [27].

Understanding the function of bystander affiliation is critical to understanding its underlying mechanism, and thus the empathic and cognitive implications of the behavior. Determining the function of bystander affiliation, however, may not be straight forward, as it seems likely to vary both across and within species. Fraser et al. [30] proposed a theoretical framework whereby the quality of the relationships between the individuals involved and the patterns of behavior expressed could determine its occurrence and function. The quality of a relationship can be thought to consist of its value (which refers to the benefits afforded by the relationship), its compatibility (the degree of tolerance within the dyad) and its security (the predictability of a partner's interactions) [31]. When a distress-reduction (consolation) function is likely, the bystander and the victim of aggression are likely to share a valuable relationship as such partners are more likely to be responsive to each other's distress [21], [30], [32]. When bystanders act as a proxy for the aggressor in repairing the opponents' relationship (‘relationship repair’ function), the bystander is likely to share a more valuable relationship with the aggressor than with the victim [24], [25], [30]. If bystander affiliation functions to protect the bystander from redirected aggression, however, the relationship between the bystander and the victim is likely to be characterized by a low degree of security and/or compatibility as those bystanders are at most risk of attack from the original conflict victim. Although this predictive framework fits the quality of relationships associated with a consoling, relationship repair or self-protection function in chimpanzees, this framework has not yet been applied to any other species.

Although the vast majority of work on bystander affiliation has been conducted on primates, and in particular on apes, bystander affiliation has recently been demonstrated in a handful of non-primate species, including dogs [33], wolves [34] and rooks [35]. As would be expected on the basis of differences in their social systems, and thus in the quality of their relationships, the patterns of post-conflict behavior across those species vary. Consistent with patterns observed in apes, reconciliation and bystander affiliation occur in dogs and wolves [33], [34], although solicited bystander affiliation was also found in these species while it may be absent in chimpanzees: [27], [36]–[38]. In contrast, rooks show patterns of post-conflict behavior that differ from any primate species as reconciliation is absent but both bystander affiliation and solicited bystander affiliation occur, although only between pair mates [35].

Here, we investigated the post-conflict behavior of ravens (*Corvus corax*), another member of the corvid family famed for their primate-like cognitive abilities [39]–[41] and complex social behavior [42]–[45]. Ravens are larger than rooks and have a comparatively longer maturation period, not reproducing until at least their third year [46], and occasionally delaying reproduction until as late as their tenth year (T. Bugnyar, unpublished data). Prior to pair-formation and the onset of territorial behavior, ravens form large non-breeder flocks during which time they may experience a broad network of social relationships [47], [48]. Recently, the value, compatibility and security of all dyadic social relationships within our captive population of ravens were ascertained [49]. This information enabled us to take advantage of the extended period during which subadult ravens have a variety of social relationships, and in particular valuable partners outside of the pair bond, to apply the predictive framework for the function of bystander affiliation [30].

As ravens live in much less stable populations than the many primate species in which reconciliation has been demonstrated and as raven sociality is characterized by a high degree of fission-fusion dynamics [50], making dispersal a more feasible and less costly option both before and after aggressive conflict, we predicted that reconciliation was not likely to be widespread and may only occur between those partners who share highly valuable relationships, for whom aggressive conflict is likely to be rare. Furthermore, the risk of renewed aggression between former opponents is likely to be high, making reconciliation too costly to occur. Consolation may thus occur as an alternative distress-alleviating mechanism. In order to find out whether ravens spontaneously provide reassurance to distressed parties, as the term consolation suggests, and to see how affiliation initiated by the bystander differs from affiliation initiated by the victim, we investigated the determinants of bystander affiliation and solicited bystander affiliation, examining in particular at quality of the bystander's relationship with the conflict opponents. We made the following predictions:

1. If bystander affiliation serves to alleviate the victim's distress (consolation), it is likely to be provided by valuable partners, as these are more likely to be responsive to each other's distress, and may occur after more intense conflicts, when the victim is more likely to be distressed [21], [30]. Solicited bystander affiliation may also alleviate the victim's distress, but empathy is not required.
2. If bystander affiliation serves a relationship repair function through mediation of a valuable partner, the bystander is likely to share a more valuable relationship with the aggressor than with the victim [25], [30]. Solicited bystander affiliation is unlikely to serve a similar function as the victim may face a high risk of aggression on approaching a bystander who shares a valuable relationship with the aggressor.
3. Bystander affiliation is predicted to serve a self-protection function if victims redirect aggression towards bystanders and the bystander-victim relationship is characterized by a low degree of compatibility and/or security, as those bystanders are most likely to be at risk of redirected aggression [26], [27], [30]. If solicited bystander affiliation occurs, it cannot fulfill the same function.
4. Finally, we predicted that if bystander affiliation or solicited bystander affiliation protects the victim from renewed attack from the aggressor, the risk of renewed aggression would be lower following the interaction than in its absence.

## Methods

### Ethical Statement

This study complied with Austrian and local government guidelines and permission was received from the Konrad Lorenz Forschungstelle to observe the ravens for this study.

### Study Subjects

We used 13 hand-reared ravens (seven males, six females) housed at the Konrad Lorenz Forschungstelle, Austria as subjects for this study. Eleven of those subjects were taken as nestlings were taken from four nests (two from zoos, two from the wild) in February 2004. The nestlings were hand-raised in their sibling groups (two males and two females, two females and one male, and two males and one female) in artificial nests, with the exception of one subject raised in a single nest with two other unrelated nestlings who were removed from the group prior to the start of this study. After fledging all the nestlings were housed together in a large aviary (ca. 240 m^2^) along with a nine-year old male and a four-year old female who were unrelated to each other or the nestlings. During the study, two subjects died as a result of predation at the end of 2004 and the two adult subjects were removed from the group in August 2005. The aviary was enriched with trees, branches, stones, tree trunks and shallow pools for bathing. The ravens were fed twice per day with meat, milk products and kitchen leftovers and always had access to water.

### Data Collection

Data were collected by TB from August 2004 to June 2006. The ravens were observed regularly throughout the day. All observed instances of aggressive conflict (defined as chase-flight, hitting or forced-retreat) were recorded. The identities of the aggressor and the victim (defined as the initial recipient of aggression) were recorded along with the intensity of the conflict (chase flight or hit = high, forced retreat = low). The post-conflict (PC)-matched control (MC) method [51] was used to collect data, where each PC was a 10-minute focal sample on the victim of aggression, recording all affiliative (defined as contact sitting, preening or beak-to-beak or beak-to-body touching) and aggressive interactions, taken immediately after the cease of aggressive conflict. MCs were similar observations taken on the same individual at the same time the next possible day. If the focal individual was involved in aggressive conflict in the ten minutes prior to the scheduled MC time, the MC was postponed for up to an hour after the time the PC was taken, or until the following day. PCs were abandoned if no MC was recorded within a week of the initial conflict.

### Data Analysis

A total of 152 PC-MC pairs were collected on 11 conflict victims (58 aggressor-victim dyads). The two adult subjects were never recorded as victims but were included in analyses involving aggressors or bystanders. For the remaining nine individuals, a mean (±S.D.) of 13.8 (±7.6) PC-MC pairs per individual were collected (range = 1–24).

#### Demonstration of Post-Conflict Interactions

Following de Waal & Yoshihara [51], for the demonstration of reconciliation PC-MC pairs were labeled ‘attracted’ if the first affiliative interaction between former opponents occurred earlier in the PC than the MC, or only in the PC. PC-MC pairs were labeled ‘dispersed’ if such affiliation occurred earlier in the MC than the PC, or only in the MC, and were labeled ‘neutral’ if affiliation occurred at the same time in the PC and the MC, or occurred in neither observation. Each PC-MC pair was similarly categorized for bystander affiliation, solicited bystander affiliation, redirected aggression and renewed aggression between the opponents. To demonstrate the occurrence of each post-conflict interaction, Wilcoxon's signed ranks tests were used to compare the proportion of attracted and dispersed PC-MC pairs at the individual level (only individuals with at least three PC-MC pairs were included in the analyses). When significant differences were found, latencies to first affiliative contact in the PC and MC periods were additionally compared using a Kaplein–Meier survival analysis with a Mantel–Cox test, which allows “censored” data (i.e. PC and MC periods in which no affiliation occurred before the end of the observation) to be taken into account. Following Call et al. [26] the triadic contact tendency (TCT) was calculated for each type of bystander affiliation for each subject as follows: (attracted pairs-dispersed pairs)/total no. PC-MC pairs.

#### When does bystander affiliation occur?

We investigated the influence of conflict intensity (high or low) and the occurrence of solicited bystander affiliation on the occurrence of bystander affiliation using generalized linear mixed models (GLMMs). A similar model investigating the effect of conflict intensity and bystander affiliation on the occurrence of solicited bystander affiliation was also run. We considered bystander affiliation or solicited bystander affiliation to have occurred when the PC-MC pair was labeled ‘attracted’ to control for baseline levels of affiliation. The identities of both conflict opponents were entered as random factors, thus controlling for variation in individual contribution to the data set. We used GLMMs with binomial error structures and a logit-link function. Akaike's information criteria (AIC) values were used to select the best (most parsimonious) model for all mixed model analyses [52]. We present only the effects of variables present in the best models.

To examine the temporal interdependency between bystander affiliation and renewed aggression, we compared the probabilities of bystander affiliation and solicited bystander affiliation occurring after and without renewed aggression and the probabilities of renewed aggression occurring after and without bystander affiliation and solicited bystander affiliation using Chi^2^ tests.

#### Which bystanders are involved?

We analyzed the effects of the quality of all potential victim-bystander dyads' relationships on the level of bystander affiliation provided and solicited bystander affiliation received to determine whether certain types of partner were more likely to be involved in bystander affiliation or solicited bystander affiliation than others. Following Fraser et al. [21], two measures of the levels of bystander affiliation and solicited bystander affiliation between partners were used, namely the consolation index (calculated as the frequency with which each subject provided bystander affiliation to each partner, divided by the frequency with which the subject was a bystander in a conflict in which that partner was a victim) and TCT values (calculated for each possible dyad). The former controls for opportunity to provide affiliation, but does not take baseline levels of affiliation between partners into account. The latter controls for baseline affiliation levels but considers the first affiliative interaction between the victim and each of the bystanders in the group, regardless of whether the victim has already affiliated with another bystander. As subsequent affiliation may function differently from the first affiliative interaction, the two measures of bystander affiliation are both necessary and complementary [21]. Linear mixed models (LMMs) were used to investigate the effects of the bystander-victim relationship on the consolation index and TCT values (run separately for bystander affiliation and solicited bystander affiliation), with the identities of the victim and the bystander entered as random variables. Predictor variables were the kinship, sex-combination, value, compatibility and security of the relationship between the bystander and the victim (see below for further explanation of these variables).

Measures of each component of relationship quality were previously obtained by entering seven behavioral variables into a principal components analysis and using the three extracted components as composite, quantitative measures of relationship value, compatibility and security [49]. The components were labeled as such as they appeared to match the characteristics proposed for value, compatibility and security by Cords & Aureli [31]. The component labeled ‘value’ consisted of strong loadings from preening, contact sitting and agonistic support. The second component, ‘compatibility’ was characterized by negative loadings for counter-intervention and aggression and a positive loading for tolerance to approaches. Variation in response to approach over time was the only significant positive loading on the final component, ‘security’. The scores provided for each dyad for each component were used as separate continuous variables in all analyses involving relationship quality in this study.

To test the hypothesis that bystanders affiliating with victims were acting as proxies for the aggressors [24], [25],we compared the qualities of the bystander-victim and bystander-aggressor relationships using LMMs. The score for the value of the bystander's relationship with each opponent for every conflict in which unsolicited bystander affiliation occurred was entered as a dependent variable, with the nature of the relationship (bystander-aggressor or bystander-victim) as predictor variable. The identities of the bystander, aggressor and victim were entered as random variables. The model was rerun with relationship compatibility and security as dependent variables. As the opponent relationship repair function though mediation of a valuable partner is likely to apply only to bystander affiliation, these analyses did not consider solicited bystander affiliation.

All analyses with the exception of GLMMs were run using SPSS v.17. GLMMs were run in R v. 2.1.0 [53] with the lme4 package [54].

## Results

### Demonstration of Post-Conflict Interactions

Although post-conflict affiliation between former opponents occurred after 16 of the 152 conflicts, no difference was found between the proportion of attracted (mean ±S.E. = 0.09±0.12) and dispersed (mean ±S.E. = 0.01±0.03) PC-MC pairs, indicating the absence of reconciliation at the group level in the study population.

**Figure 1:**
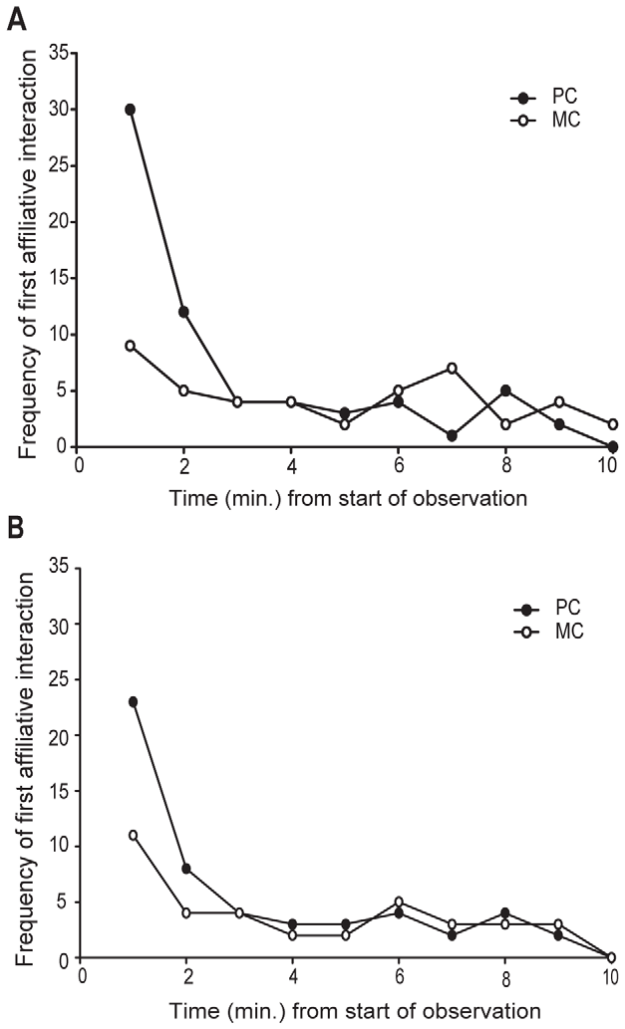
Demonstration of bystander affiliation and solicited bystander affiliation in ravens. Frequency distributions of latency to first affiliative post-conflict interaction directed from a bystander to the conflict victim (A) and directed from the victim to a bystander (B) in post-conflict periods (PCs; filled circles) and matched control periods (MCs; open circles).

For bystander affiliation, the proportion of attracted (mean ±S.E. = 0.38±0.06) PC-MC pairs was significantly higher than the proportion of dispersed (mean ±S.E. = 0.15±0.04) PC-MC pairs (Wilcoxon: N = 10, T = 50, P = 0.020). A survival analysis confirmed the significant tendency for affiliation from a bystander to the conflict victim to occur earlier in the PC than in the MC (Kaplan-Meier Survival Analysis: Mantel Cox test: N = 152 PC-MC pairs, Chi^2^ = 12.198, P<0.001; Figure 1a), demonstrating the occurrence of bystander affiliation in ravens. Mean (±S.D.) individual TCT for bystander affiliation was 0.206 (±0.266).

For solicited bystander affiliation, the proportion of attracted (mean ±S.E. = 0.27±0.03) PC-MC pairs was also significantly higher than the proportion of dispersed (mean ±S.E. = 0.14±0.04) PC-MC pairs (Wilcoxon: N = 8, T = 33, P = 0.039). A survival analysis confirmed the significant tendency for affiliation from a victim to a bystander to occur earlier in the PC than in the MC (Kaplan-Meier Survival Analysis: Mantel Cox test: N = 152 PC-MC pairs, Chi^2^ = 5.410, P = 0.020; Figure 1b), demonstrating that solicited bystander affiliation also occurs in ravens. Mean (±S.D.) individual TCT for solicited bystander affiliation was 0.21 (±0.31).

We found no significant difference between the proportion of attracted (mean ±S.E. = 0.30±0.08) and dispersed pairs (mean ±S.E. = 0.17±0.04) for redirected aggression (Wilcoxon: T = 47, N = 11; P = 0.229), indicating that victims were no more likely to attack bystanders after losing a conflict than during control periods. Conversely, for renewed post-conflict aggression between opponents, the proportion of attracted pairs (mean ±S.E. = 0.28±0.09) was significantly higher (Wilcoxon: T = 39.5, N = 9; P = 0.043) than the proportion of dispersed pairs (mean ±S.E. = 0.07±0.03), and renewed aggression was likely to occur earlier in the PC than the MC (Kaplan-Meier Survival Analysis: Mantel Cox test: N = 152 PC-MC pairs, Chi^2^ = 30.081, P<0.001) indicating that victims were at risk of renewed aggression from the original aggressor during the post-conflict period.

### When does bystander affiliation occur?

**Figure 2:**
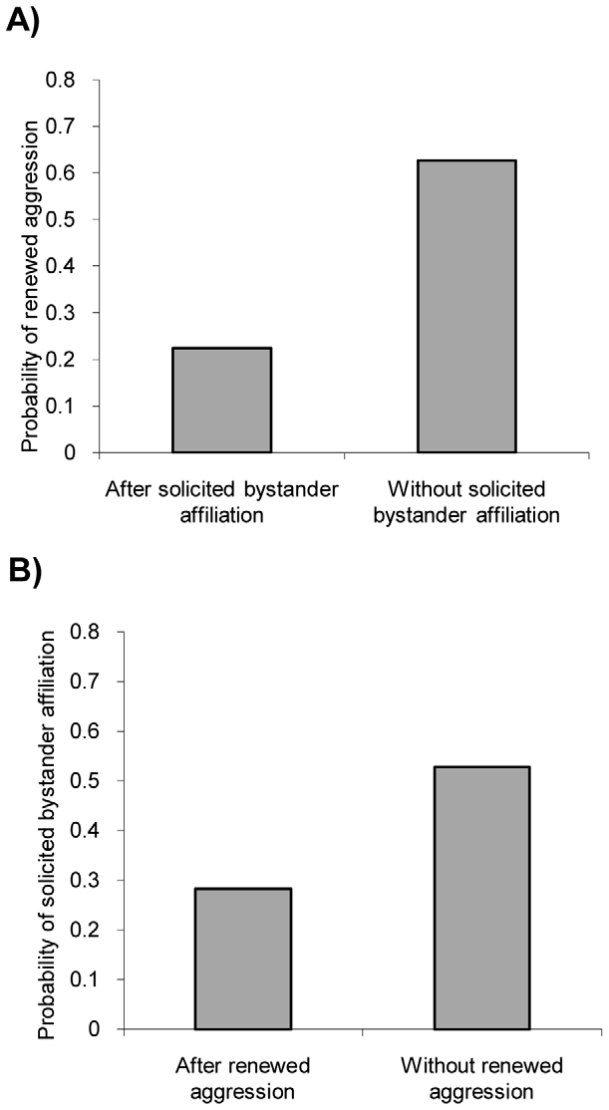
The interdependency of solicited bystander affiliation and renewed aggression between former opponents in ravens. *P = <0.005

Bystander affiliation, but not solicited bystander affiliation, was more likely to occur after conflicts characterized by a higher intensity of aggression (Table 2). Bystander affiliation and solicited bystander affiliation significantly predicted each other's occurrence (Table 2). We found no temporal interdependency between bystander affiliation and renewed aggression, as renewed aggression was not more likely to occur after bystander affiliation than alone (χ^2^ = 2.063, df = 1, P = 0.151) and bystander affiliation was not more likely to occur after renewed aggression than alone (χ^2^ = 2.465, df = 1, P = 0.1164). In contrast, renewed aggression was less likely to occur after solicited bystander affiliation than alone (χ^2^ = 8.551, df = 1, P = 0.004; Figure 2a) but solicited bystander affiliation was not more likely to occur after renewed aggression than alone (χ^2^ = 2.057, df = 1, P = 0.152; Figure 2b).

**Table 2:** Results of the best model from the GLMM investigating the effect of conflict and post-conflict variables on the occurrence of bystander affiliation and solicited bystander affiliation (BA).

Dependent variable	Predictor Variables	β	S.E.	z	P
Bystander Affiliation	Solicited Bystander Affiliation	1.409	0.399	3.529	<0.001
	Intensity	1.333	0.575	2.319	0.020
Solicited Bystander Affiliation	Bystander Affiliation	1.372	0.376	3.653	<0.001

Victim and aggressor identities were included as random factors.

### Which bystanders are involved?

When the consolation index was used as a measure of bystander affiliation or solicited bystander affiliation, such interactions were most likely to occur between partners who shared valuable relationships (LMM: bystander affiliation: β = 0.093, S.E. = 0.017, t = 5.430, P<0.001; solicited bystander affiliation: β = 0.075, S.E. = 0.011, t = 7.147, P<0.001). However, when baseline levels of affiliation were controlled for using TCT values, only kin were more likely to engage in post-conflict affiliation with the victim (bystander affiliation: β = 0.059; S.E. = 0.018; t = 5.430; P = 0.002; solicited bystander affiliation: β = 0.078; S.E. = 0.013; t = 5.871; P<0.001).

**Figure 3:**
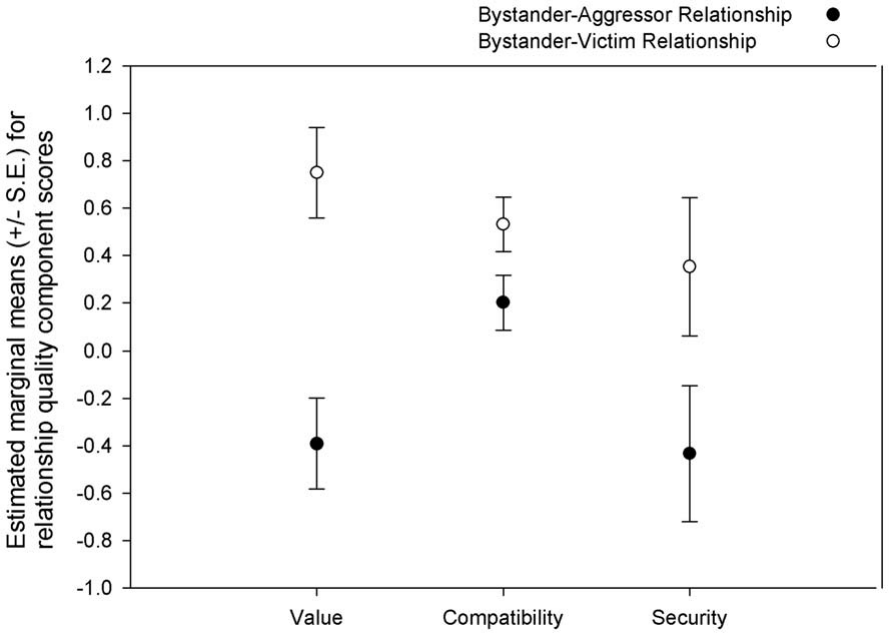
The quality of bystander-aggressor and bystander-victim relationships in ravens. Results of LMM analyses comparing components (value, compatibility and security) of the bystander's relationships with the aggressor and the victim when post-conflict affiliation from a bystander to the conflict victim occurs.

Bystanders who initiated post-conflict affiliation with victims of aggression shared more valuable (β = 1.141; S.E. = 0.086; t = 13.203; P<0.001), more compatible (β = 0.329; S.E. = 0.049; t = 6.703; P<0.001) and more secure (β = 0.787; S.E. = 0.220; t = 3.583; P<0.001) relationships with the victim of the conflict than with the aggressor (Figure 3).

## Discussion

The occurrence of reconciliation could not be confirmed in this group of ravens, consistent with findings in rooks [35]. Reconciliation has been shown to repair the opponents' relationship and reduce post-conflict distress [4], [55], and is thus considered to be the preferred post-conflict interaction in terms of mitigating the costs of aggressive conflict [6]. However, reconciliation should still only occur when its benefits outweigh the costs. Victims were at higher risk of renewed aggression in post-conflict than matched-control periods, suggesting that the risks of renewed aggression upon reconciliation may be too high.

In contrast to reconciliation, both bystander affiliation and solicited bystander affiliation were demonstrated as post-conflict interactions in ravens. Bystander affiliation was more likely to occur after more intense conflicts, which, as victims may experience a higher degree of distress following more intense conflicts, suggests that bystander affiliation may indeed serve a distress-alleviating, or consoling, function. Furthermore, bystanders who provided post-conflict affiliation were likely to share a valuable relationship with the victim of aggression, supportive of a distress-alleviating function as such partners are more likely to be responsive to each other's distress [56], an effect even more likely for kin. Our results are consistent with previous research showing that consolation in chimpanzees is provided by kin and other valuable partners [21], [32].

Sharing a valuable relationship with the victim does not, however, necessarily rule out the possibility that the bystanders also share a valuable relationship with the aggressor, and thus bystanders may still be acting as proxies for the aggressor in reconciling the opponents. For this to be the case bystanders would be expected to share a more valuable relationship with the aggressor than with the victim [25], [30]. Our findings show that bystanders shared more valuable, more compatible and more secure relationships with the conflict victim than with the aggressor, evidence that in ravens opponent relationship repair through mediation of a valuable partner is an unlikely function for bystander affiliation.

The fact that bystanders shared a valuable relationship with the victim, and that their relationship was no less compatible or secure than the victim's relationship with non-affiliating bystanders lead us to reject the hypothesis that bystanders affiliate with the victim of aggression to protect themselves from redirected aggression, as such bystanders are unlikely targets [30]. Furthermore, as redirected aggression could not be demonstrated as a post-conflict interaction, bystander affiliation is unlikely to serve a self-protection function in this group of ravens.

Interestingly, in chimpanzees, the only species in which consolation has been shown, most studies found that solicited bystander affiliation did not occur [27], [36]–[38], [57]. Conversely, we found not only that solicited bystander affiliation occurs in ravens, but that it is directed towards the same bystanders (valuable partners) who are likely to direct post-conflict affiliation towards victims. Furthermore, when one form of bystander affiliation occurred, the other was also likely to occur. However, the fact that aggression was less likely to occur after solicited bystander affiliation, but not unsolicited bystander affiliation, is suggestive of differing functions for the two interactions. The reduced risk of renewed aggression after solicited bystander affiliation suggests that victims may affiliate with bystanders in order to protect themselves from further attack.

According to the predictive framework, our findings are consistent with a distress-alleviating function for bystander affiliation and should thus be considered to be consolation. The term ‘consolation’, however, infers not only the function of the interaction, alleviating the victim's post-conflict distress, but also its mechanism, empathy for the distressed victim. That bystander affiliation was more likely to occur after intense conflicts, when victims were more likely to be distressed, and that it was most likely to be provided by valuable partners, are supportive of both the functional and mechanistic components of consolation. As emotional contagion (when a subject's emotional state reflects the state perceived in a partner [7], [11]) forms the core basis of empathy, it seems likely that potential consolers would be more likely to respond the perception of increased distress. Moreover, empathy is promoted by close social bonds [11], [58], [59], consistent with our finding that bystander affiliation was provided by bystanders with whom the victim shared a valuable relationship. That kin (a subset of valuable partners) were most likely to console the victim further increases support for ravens' emotional sensitivity to others, as predictions for the occurrence of empathy are consistent with kin selection theory [7].

Whether the initiator of post-conflict affiliation between a bystander and a victim is the bystander or the victim is a critical differentiation when a consoling function is considered because while both interactions may alleviate the victim's distress, only affiliation initiated by the bystander is likely to require empathy. However, if consolation provided by a bystander is preceded by a vocal or other signal from the victim ‘requesting’ support, such a cognitive ability may not be necessary. Thus, although we found suggestive evidence for different functions for bystander affiliation and solicited bystander affiliation, caution must always be taken when interpreting the initiator of an interaction, as signals prior to the first physical interaction may go undetected. Notably, vocalizations were not recorded during this study, and are not usually taken into account in studies of post-conflict behavior (exceptions: [24], [60]), despite the role that they may play in the facilitation of physical affiliative interactions.

All studies on consolation thus far have, for methodological reasons, focused on the effect of consolation on the victim rather than on the consoler. In order to fully understand the mechanism behind consolation, however, we really need to understand more about the consequences of offering consolation for potential consolers. Firstly, although bystanders may experience post-conflict distress [61], we do not know whether consolation alleviates the consoler's as well as the victim's distress. Although empathy may be involved either way, if consolation alleviates the consoler's distress, it may occur as a result of ‘personal distress’, (self-centered distress born from empathy with another's distress [7]) rather than ‘sympathetic concern’ (concern about another's state and attempts to ameliorate this state), which relies on the separation of internally and externally generated emotions. Secondly, if providing consolation entails a risk of aggression for the consoler, the costs of such an act suggest that the consolers' behavior is altruistic. Such ‘directed altruism’ implies an underlying mechanism of sympathetic concern [7]. Although we were not able to analyze the relative increase in risk of aggression that a bystander faces when consoling a victim, in six out of 64 cases of consolation (9.4%), the consoler was subsequently attacked (five times by the aggressor, once by another bystander) within the post-conflict period. In one additional case, a potential consoler (a valuable partner of the victim) was attacked by the aggressor after approaching the victim, but before consolation could take place. It seems likely, therefore, that providing consolation is not risk-free, and may thus be altruistic.

The patterns of post-conflict behavior observed in ravens match what we would expect from what we know about the structure of their relationships. As a pair-bonded species, adult ravens are likely to share valuable relationships primarily with their mates, and thus patterns of post-conflict behavior among adults are expected to resemble those described in rooks [35], where post-conflict bystander affiliation occurs only within pairs and reconciliation is completely absent. However, sub-adult ravens form large non-breeder flocks [47], [48] and actively recruit others to feeding sites [62], conferring a competitive advantage at monopolizable food sources when competing with territorial pairs [63]. Thus, sub-adult ravens may cultivate valuable relationships with a greater number of individuals [49], which may be reflected in their conflict resolution strategies. In this study, patterns of post-conflict behavior suggested that bystanders consoled victims with whom they shared valuable relationships, indicating that the ravens may employ strategies similar to those used by chimpanzees to alleviate distress and mitigate the costs of aggressive conflict. Furthermore, our findings are consistent with the idea that ravens may show similar expressions of empathy for valuable partners. More research is needed to understand the consistency of patterns of raven post-conflict behavior across populations and developmental periods and how transferable such patterns observed in aviary-housed ravens are to wild ravens. Nevertheless the findings of this study represent an important step towards understanding how ravens manage their social relationships and balance the costs of group-living. Furthermore, they suggest that ravens may be responsive to the emotional needs of others.
